# Optimal Design for Compliant Mechanism Flexure Hinges: Bridge-Type

**DOI:** 10.3390/mi12111304

**Published:** 2021-10-23

**Authors:** Chia-Nan Wang, Fu-Chiang Yang, Van Thanh Tien Nguyen, Quoc Manh Nguyen, Ngoc Thai Huynh, Thanh Thuong Huynh

**Affiliations:** 1 Department of Industrial Engineering and Management, National Kaohsiung University of Science and Technology, Kaohsiung 80778, Taiwan; cn.wang@nkust.edu.tw; 2 Industrial University of Ho Chi Minh City, Nguyen Van Bao Street, Ward 4, Go Vap District, Ho Chi Minh City 70000, Vietnam; 3 Faculty of Mechanical Engineering, Hung Yen University of Technology and Education, Hung Yen 16000, Vietnam; 4 Department of Mechanical Engineering, Campus II, Can Tho University, Can Tho 94000, Vietnam; thanhthuong@ctu.edu.vn

**Keywords:** optimization design, compliant mechanism, grey-based Taguchi method, artificial neural network

## Abstract

Compliant mechanisms’ design aims to create a larger workspace and simple structural shapes because these mechanical systems usually have small dimensions, reduced friction, and less bending. From that request, we designed optimal bridge-type compliant mechanism flexure hinges with a high magnification ratio, low stress by using a flexure joint, and especially no friction and no bending. This joint was designed with optimal dimensions for the studied mechanism by using the method of grey relational analysis (GRA), which is based on the Taguchi method (TM), and finite element analysis (FEA). Grey relational grade (GRG) has been estimated by an artificial neural network (ANN). The optimal values were in good agreement with the predicted value of the Taguchi method and regression analysis. The finite element analysis, signal-to-noise analysis, surface plot, and analysis of variance demonstrated that the design dimensions significantly affected the equivalent stress and displacement. The optimal values of displacement were also verified by the experiment. The outcomes were in good agreement with a deviation lower than 6%. Specifically, the displacement amplification ratio was obtained as 65.36 times compared with initial design.

## 1. Introduction

For over a decade, numerous scientists and researchers have investigated several kinds of flexure hinges to use as the traditional joints. Yong and Lu investigated the kinetostatic model with a 3-RRR compliant mechanism [1]. These joints could be used as rotational joints for a 3-DOF (degrees of freedom) parallel mechanism with smooth and high-precision motion in micro/nanomanipulation work, which Tian et al. [2] presented in their recent studies. Qi et al. showed a displacement-amplification bridge-type mechanism, referring to the elastic beam theory (EBT) [3] and kinematics. The equivalent formula and FEM (finite element method) to analyze failures in Triple-LET and LET flexure hinges were employed by Qiu, Yin, and Xie [4]. Tian et al. used the finite element method to simulate filleted V-shaped flexure hinges and compared them with closed-form compliance equations [5]. Yang et al. used super-elastic materials to produce a bending hinge, and their calculations and numerical experiments were able to accurately predict displacement and reduce computational costs more efficiently than ANSYS FEA [6]. Xu et al. developed DAR employing the Euler-Bernoulli of EBT and confirmed its findings using finite element analysis and conducting experiments [7]. Liu and Yang introduced a new approach applying EBT to investigate the effect of the displacement amplification ratio (DAR) external loads, and the results were validated using the FEM [8]. The law of conservation of energy and EBT improved the methodology of the mechanisms corresponding to the spheres and rhombuses, and the results were compared to those obtained by the FEM and the conducted experiments, as employed by Ling et al. [9]. Choi et al. studied and demonstrated a compatible mechanism with the type of bridge, fully respecting the concentrated and distributed force [10]. Their results have been confirmed by previous experiments and studies. Ma et al. note that DAR increases with the decreasing thickness of the flexible joint, and this problem has been investigated using the FEM and mathematical modeling [11]. A prefabricated modular static modeling tool was introduced by Ling et al. for analyzing and designing the wide range of flexible joints, which is used in the precision-positioning phase. The results of this approach were compared with those of the FEM and its earlier studies [12].

In 2018, Ling et al. presented their findings regarding a semi-analytical finite element approach for complex compliant mechanisms by using Lagrange’s equation [13]. Sabri et al. performed experiments to measure the displacement of silicon XY-micro-stages [14]. A new pseudo-rigid-body model of a flexure hinge was proposed by Šalinic et al. [15]. The concept of virtual operation created a matrix relationship that is used to determine the quasi-static responses of a compatible mechanism due to external loads. Lai et al. used two L-shaped levers and a spherical mechanism to eliminate bending moments and shear forces [16]. The stiffness matrix was used for identifying DAR. It was confirmed by FEA and the conducted experiments. For highly accurate tracking and positioning, Wang and Zhang developed a compact flat nano-localization platform with three free capabilities, in which three two-level switchable amplifiers are symmetrically positioned to obtain high magnification [17,18,19,20,21], and this was determined by the experiments.

The consistency mechanism for all types of bridges described above is the consistency mechanism for types of bridges that use flexible hinges. The presence of stiffness in the four arms of this rectangular mechanism is a serious weakness in the mechanism frequency [19,22,23,24,25,26]. In comparison with the other bridge-type mechanisms, the distributing one is suitable for flexible multi-beam parts for increasing the resonant frequency of the mechanism, instead of notched hinges and rigid bodies. The compliant mechanisms could meet the demand for longer lifetime and better performance, in comparison with dynamic mechanical amplifiers used in the past. In contrast, the adaptation mechanism’s mechanical property of the bridging distribution has never been investigated. At the design step, a sufficiently easy analytical model makes it possible to define the structural factors regarding the performance demands expected from behaviors of the mechanism [27,28,29,30]. In the present study, the mechanical properties of the deformation (displacement) of the bridge-type mechanism have been found and analyzed in detail. The authors use the stiffness matrix method; therefore, the input stiffness used to predict the magnification of the theoretical displacement is confirmed by the FEA. Comparison of the analytical model with the results of the FEA shows that the analytical model has higher precision. According to the analyzed modeling, the influences of shape and material factors on the performance of the bridge-type mechanism, such as displacement ratio and stiffness, have been analyzed.

The motivations of this work are a project that optimizes the design parameters in the bending hinge DAR of the bridge-matching mechanism using gray relational analysis based on the Taguchi method [31,32,33,34,35], FEM in ANSYS, and artificial neural networks [36,37,38,39,40,41]. A gap is often present in many kinds of classical joints, leading to friction and vibration, causing the wear of the joints. Flexure hinges were developed to eliminate the gap, and their effects have been applied in many popular mechanisms. In this study, the optimal design for bridge-type compliant mechanism flexure hinges has been conducted and investigated.

The contribution of this study is to analyze the gateway types of distributed compliance mechanisms. The compliant distributed bridge mechanism has distributed stress and low quality and has a longer life and superior performance compared to the traditional mechanical swing arm pivot-based amplifier. We use the stiffness matrix method to generate an analysis model and predict the input stiffness by comparing the displacement gain of the bridge-type mechanism. For verifying the analysis modeling mechanism, the FEA method of the bridge mechanism has also been performed via the ANSYS Workbench.

## 2. Developed Modeling and Applied Finite Element Method (FEM)

### 2.1. Studied Compliant Mechanism—A Developed Model

**Figure 1:**
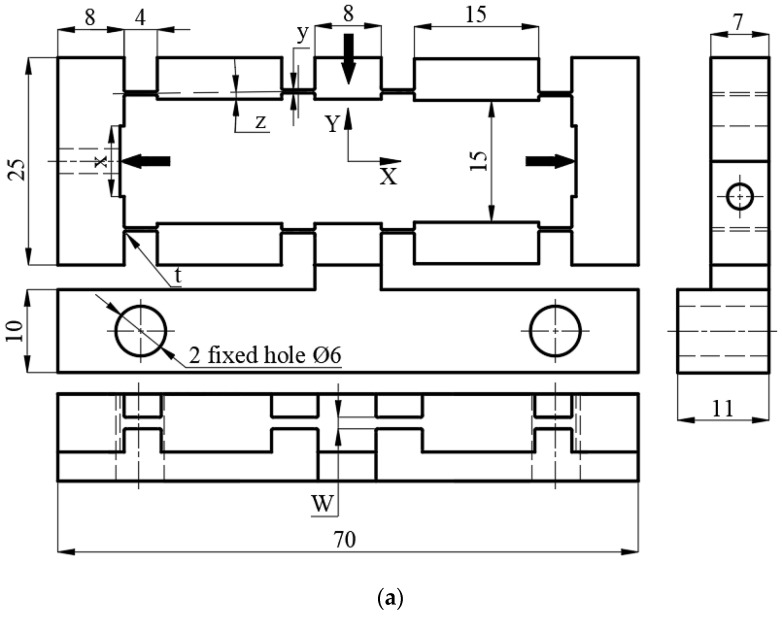

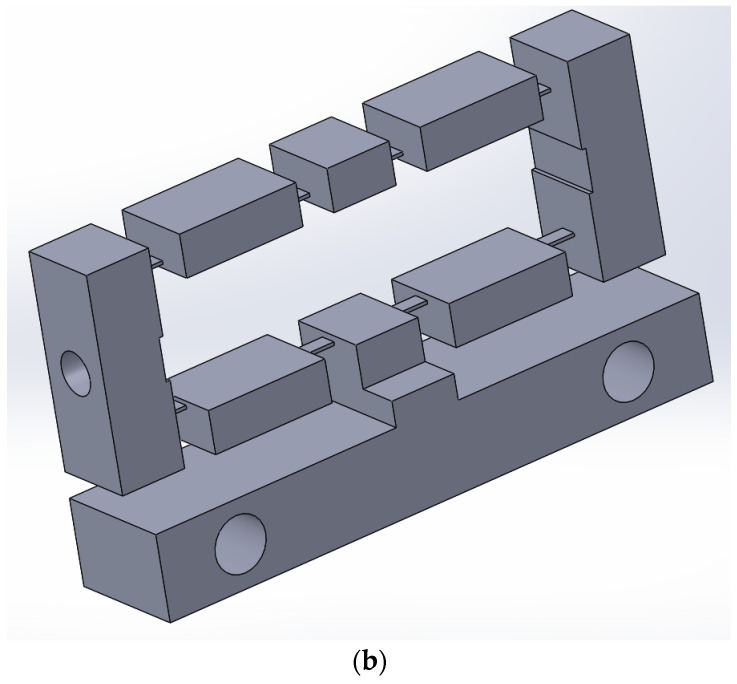
Developed compliant mechanism model of bridge-type: (**a**) 2D drawings, and (**b**) 3D drawing.

Figure 1 presents our developed compliant mechanism. Its primary dimensions are 70, 25, and 10 mm. Figure 1a shows the 2D drawings, and Figure 1b shows the 3D model. The mechanism has eight bendable joints of 4 mm long and with variable thickness, four middle cases, two inlet cases with variable inlet length, one fixed case, and one outlet case of 8 mm long and 10 mm wide. Absolute force, distribution force, or displacements are employed as inlet parameters to the compliant mechanisms, and the outlet frame moves from top to bottom on the *Y*-axis.

### 2.2. Analyzing the Finite Element Method (FEM)

**Figure 2:**
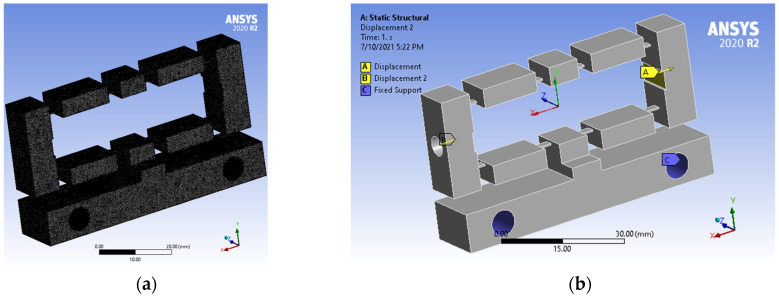
(**a**) Modeling meshes, and (**b**) locations of input displacements and fixed supports.

The material used for the developed compliant mechanism is aluminum. First, the developed mechanism model was built employing the SOLIDWORK software and then imported into the statically structural environment (ANSYS tools). It is clear to see that the mechanical material of this developed model is AL-7075 (aluminum). The mechanism meshes are presented in Figure 2a. Fixed support is utilized for fixing surface A. The double-input bodies with 0.01 mm of each (displacement) were placed on the B-surface and C-surface, see Figure 2b.

## 3. Investigative Methodology

### 3.1. Grey Relational Analysis Based on Taguchi Method

The Taguchi method (TM) in Minitab 18 software was applied to create an orthogonal array: the optimal output characteristics obtained from the theory model must be pointed out first, and then the optimal methods are applied. However, the deviations compared with the theoretical modeling were huge, and therefore, the optimal methods could not be approved. Thus, in this investigation, we applied TM based on grey relational analysis [18,19,20,21,22,23,24,25,26,27] to optimize these output characteristics.

Step 1: Choosing optimization combination parameters for the output characteristics.

Step 2: Defining the primary control factors with their specific levels.

Step 3: Laying out (L27) the orthogonal array.

Step 4: Carrying out numerical computation and collected numerical results.

Step 5: Employing the grey relational analysis (GRA), which is known as the method for comparing the alternative values of a system undertaking analysis to calculate the significance of the design variable. The GRA method is employed to discretize the frequency. GRA was carried out as below.

Normalizing: Rewrite every single sequence from 0 to 1.

- GRC stands for grey relational coefficient (*γ*). This employs a quantitative methodology. GRC is required initially. After that, we determined the grey relational grade (GRG).
- Estimation of the normalized coefficient.
- Determination of the entropy.
- Computation of the sum of entropy values.
- Determination of the weight.

Step 6: Analysis of the S/N ratio: larger-the-better methodology [28,29,30,31,32,33,34].

Step 7: Analyzing the regression equation.

Step 8: Analysis of variance.

Step 9: Analysis of mean and predicted outcomes.

Step 10: Predicted GRG by using an artificial neural network.

Step 11: Verify results.

$F_{\alpha }(1,f_{e})$ values can be found in reference [35].

In this paper, the software program Minitab 18 was used to create the TM, for the S/N evaluation, and for the evaluation of ANOVA [41,42,43,44,45,46]. In particular, acquired outcomes are presented in Section 4 in this paper.

### 3.2. Stage of Artificial Neural Network (ANN)

In this study, we used 3 layers: the input layer with 5 enter parameters, the hidden layer with 11 neurons, and 1 output layer with 1 output neuron. The community becomes educated on the usage of the Levenberg–Marquardt hybrid (trainlm) [36,37,38,39,40,41,42,43,44]. The shape of the ANN was offered in [27] and other design method suggestions of structures were mentioned in [47,48,49,50]. The inlet body length, angle of incline, thickness, the radius of the fillet, and the width of the (flexure) hinges were used as the inlet factors. In the contract, displacement and pressure were used as the outlet factors. The numerical values were applied for training. The numerical values then were applied for testing.

### 3.3. Statistical Analysis

◾ The model accuracy was evaluated utilizing 4 error standards, which are listed in [51].
◾ RMSE stands for the root mean squared error, which differs from forecast values to the numerical ones or observed actual ones.
◾ MSE stands for the mean square error, which is the square value of the root mean square.
◾ MAPE stands for the mean absolute error percentage.
◾ *R*^2^ stands for the determination coefficient, which has to be at least 0.8 for forecast models to be accepted:
  (1) $$R^{2}=1-\frac{\sum_{i=1}^{m}{\left(x_{i}-y_{i}\right)}^{2}}{\sum_{i=1}^{m}{\left(x_{i}-{\bar{y}}_{i}\right)}^{2}}$$
  where *m* stands for the range of experimental simulations, $x_{i}$ and $y_{i}$ represent the numerical and forecast values respectively, and ${\bar{y}}_{i}$ represents the mean of the numerical value.

## 4. Primary Results and Detailed Discussion

### 4.1. Simulation Plan

The parameters and their levels are listed in Table 1 in detail. Thereby, variable *x* is the length of the input frame, which changes between 5 and 10 mm (and 15 mm). The variable *y* is the flexure hinge thickness, changing between 0.4 and 0.6 mm (and 0.8 mm). The variable *z* is known as the angle of the incline in the two-flexure hinges, changing between 0.7 and 1 degree (and 1.3 degrees). The variable *t* is known as the flexure hinge fillet radius, and changes between 0 and 0.2 mm (and 0.4 mm). The variable *w* is the width of the flexure hinge, alternating between 4 and 6 mm (and 8 mm).

By using Minitab 18.0, the numerical results of the generated output displacement values and equivalent stress values are presented in Table 2. Besides, the orthogonal arrays were created. The finite element analysis in ANSYS revealed that the design variable significantly affected displacements and stresses.

$D_{i}^{*}(1)\;and\;D_{i}^{*}(2)$ are objective functions, and the values are presented in Table 3. For the bridge-type compliant mechanism to work optimally, large displacement is better, and small stress is better. The values *Δ_oi_*(1) and *Δ_oi_*(2) are considered as the displacement and stress deviation values respectively, which are presented in Table 3. The GRC values *γ_i_*(1) and *γ_i_*(2) represent displacement and stress respectively, and GRG (*ψ_i_*) is calculated and ranked, as shown in Table 4. The sixth column in Table 4 illustrates the results of the signal-to-noise (S/N) analysis, the seventh column outlines the predicted values of GRG by using ANN, and the eighth column is the error between the predicted values of ANN and the simulation values. The predicted values of GRG by using ANN and the simulated values by using ANSYS are in good agreement. The error is low, as shown in Table 5.

### 4.2. S/N Ratio Analysis

**Figure 3:**
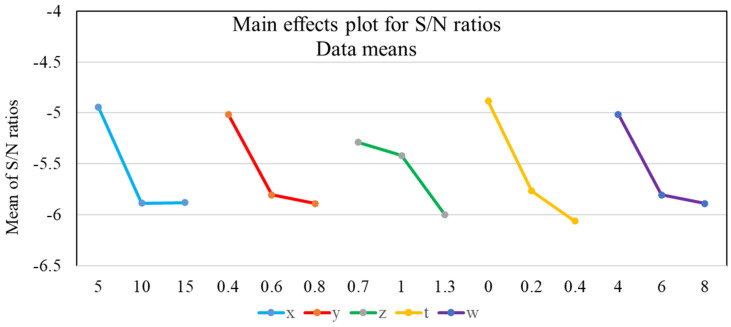
The results of the S/N analysis.

The outcomes of the analysis of the signal-to-noise ratio are listed in Table 5 and used to draw the plot of the S/N analysis as shown in Figure 3. The maximum value of the mean of S/N indicates which optimal level of the design variable (*t*) needs to be investigated and evaluated. Thereby, the optimal levels of design variables *x*, *y*, *z*, *t*, and *w* were selected (5 mm, 0.4 mm, 0.7 degrees, 0 mm, and 4 mm, respectively), corresponding with *x*1*y*1*z*1*t*1*w*1. The slope of the graph identifies that the larger the slope of the variable is, the more strongly the variable affects GRG. Therefore, according to Figure 3, variable *y* is the strongest. The variables of *t*, *x*, *w*, and z eventually decrease. The problem was ranked as shown in Table 4.

**Figure 4:**
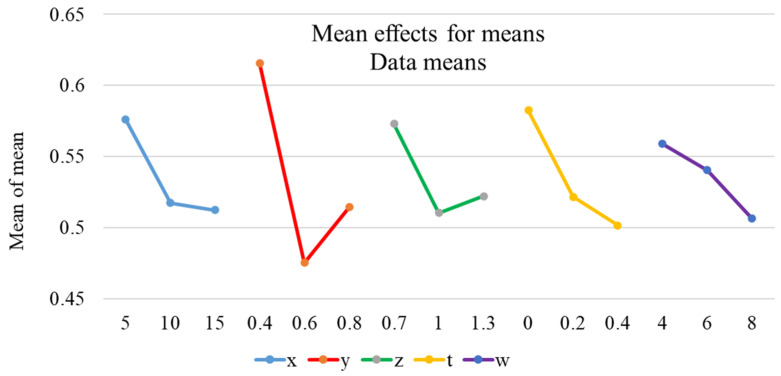
The result of mean analysis.

The outcomes of the analysis of the mean values are listed in Table 6 and used to draw the plot of the analysis of the mean values as shown in Figure 4. The maximum mean value of GRG indicates that it is the optimal level for the design variable. Thereby, the optimal levels of the design variables (*x*, *y*, *z*, *t*, and *w*) are 5 mm, 0.4 mm, 0.7 degrees, 0 mm, and 4 mm respectively, corresponding with *x*1*y*1*z*1*t*1*w*1. The slope of the graph identifies that the larger the slope of the variable is, the more strongly the variable affects GRG. Therefore, according to Figure 4, variable y is the strongest.

### 4.3. ANOVA for Output Response

The ANOVA outcomes are presented in Table 7. The first column is the design variable, regression equation (RE), error, and total. The second column is the degree of freedom of the RE, design variable, and error. The fourth column presents the contribution of the design variable. The results demonstrate that the design variables significantly affected displacement and stress and are in good agreement with the S/N analysis, the mean analysis, and the finite element analysis in ANSYS. Since the *p*-values are less than 0.005 and the F-values are higher than 2, it is clear to see that the *R*-*square* is 96.61%, *R*-*square* (adj) = 94.76%, and *R*-*square* (pred) = 92.92%, respectively.

### 4.4. Regression Analysis

**Figure 5:**
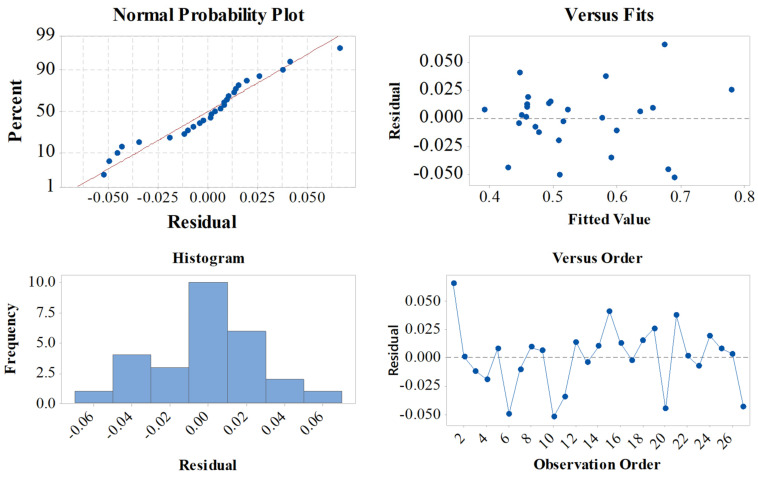
Residual plot for GRG.

The predicted results were achieved the regression analysis of GRG and presented as the residual graph for GRG in Figure 5. In this study, the normal probability plots demonstrate that the simulation data and predicted data by RE are approximated to each other, and the interval error is between −0.045 and 0.045. The interval was also verified by Equation (2):

(2) $$\begin{array}{l} GRG=(1.1054+0.01869x-1.056y-0.0791z+0.251t-0.01667w\\ +0.00072x^{2}+1.345y^{2}-0.0598xy-0.0598yt{)}^{2} \end{array}$$

**Figure 6:**
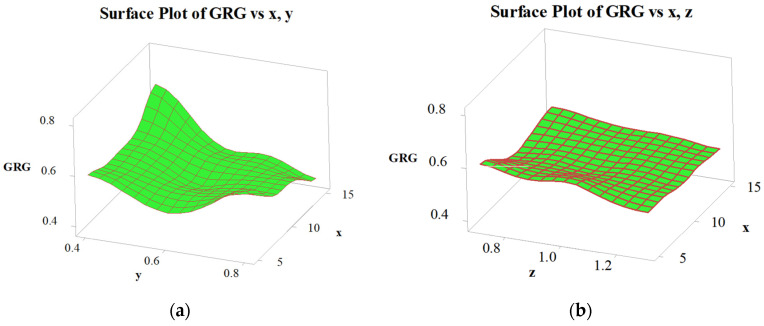

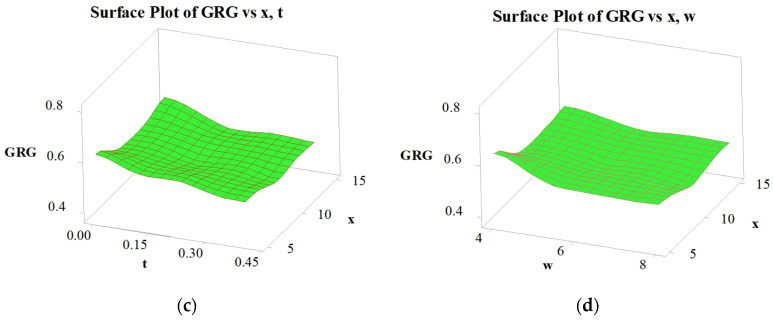
Surface plot for GRG. (**a**) Surface plot of GRG with *x*, *y*; (**b**) Surface plot of GRG with *x*, *z*; (**c**) Surface plot of GRG with *x*, *t*; (**d**) Surface plot of GRG with *x*, *w*.

In Figure 6, the surface plot for GRG identifies that the design variables have significantly changed the GRG values. The analytical outcomes are in good agreement with the results of the S/N analysis, the FEM results, the ANOVA results, and the predicted results of the regression analysis.

### 4.5. Artificial Neural Network

**Figure 7:**
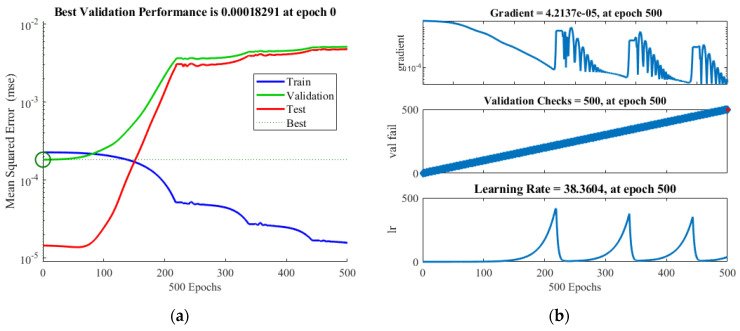
Relationship between simulation values and ANN model values for displacement. (**a**) the best validation performance at epoch 0; (**b**) Gradient at epoch 500.

The simulation results were utilized for comparison with those of the ANN model values. The performance plots are shown in Figure 7 for GRG. The best validation performance was 0.00018291 at epoch 0.

**Figure 8:**
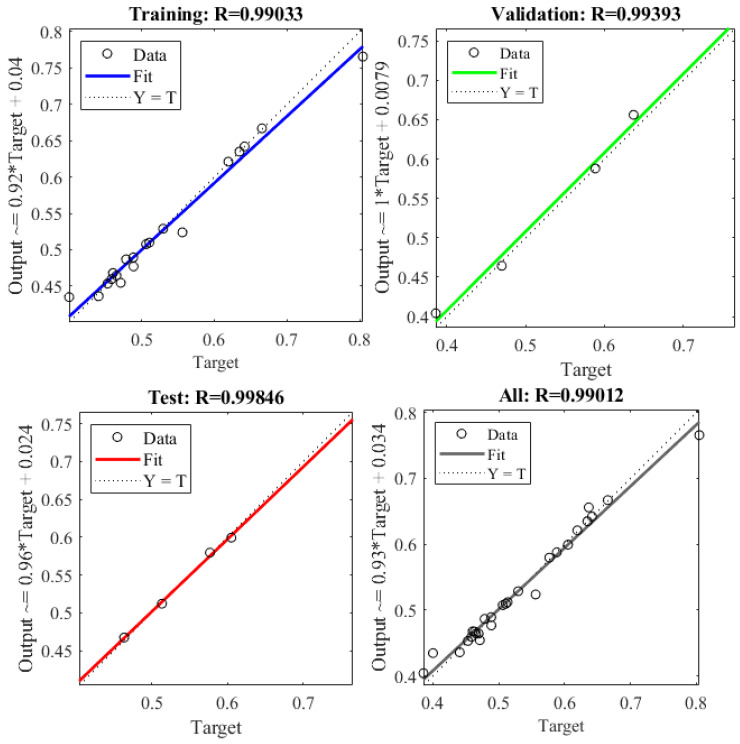
Statistical analysis of GRG.

The results of statistical analysis of GRG are presented in Table 8, and the results showed that RMSE, MSE, MAPE, and *R*-square (see Figure 8) were 0.01718171, 0.000295199, 2.109164529, and 0.998937275, respectively. The values are very low. This problem proves that the predicted values of GRG of the ANN method are in good agreement with the values of GRG achieved from the GRA based on the FEM.

The predicted value of GRG by the Taguchi method $\left(\mu_{G}\right)$ was obtained as follows:

$$\mu_{G}=G_{m}+\sum_{i=1}^{q}(G_{0}-G_{m})=x1+y1+z1+t1+w1-4G_{m}$$

It can be seen that the values *x*1, *y*1, *z*1, *t*1, and *w*1, as listed in Table 6, were 0.5759, 0.6157, 0.5371, 0.5826, and 0.5589, respectively. The GRG mean value (*G_m_*) was 0.5353.

$$\mu_{G}=0.5759+0.6157+0.5731+0.5826+0.5589-4\times 0.5353=0.765$$

A 95% confidence interval (*CI*) was gained (see Table 9) utilizing:

$${CI}_{CE}=\pm \sqrt{F_{\alpha }(1,f_{e})\times Ve\times \left[\frac{1}{R_{e}}+\frac{1}{n_{eff}}\right]}=\pm \sqrt{4.4513\times 0.000329\times (\frac{1}{\frac{27}{1+10}}+1)}=\pm 0.045$$

$$0.72<\mu_{confirmation}<0.81$$

where, *Ve* = 0.000329, $F_{\alpha }(1,f_{e})=F_{0.05}(1,17)=4.4513$ [35], $n_{eff}=\frac{27}{1+10}$, $R_{e}=1$.

Table 9 presents a comparison of the results among the predicted values and optimal values of the Taguchi method, ANN, and RE. These results prove that the predicted and optimal values of the three methods are in good agreement, with errors of less than 4%.

**Figure 9:**
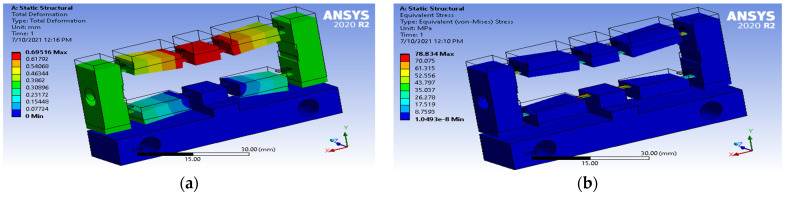
The optimal results. (**a**) The optimal result of displacement; (**b**) The optimal result of stress.

The optimal values of displacement and equivalent stress were obtained as 0.69516 mm and 78.834 MPa respectively, as depicted in Figure 9.

### 4.6. Verified Experiment

**Figure 10:**
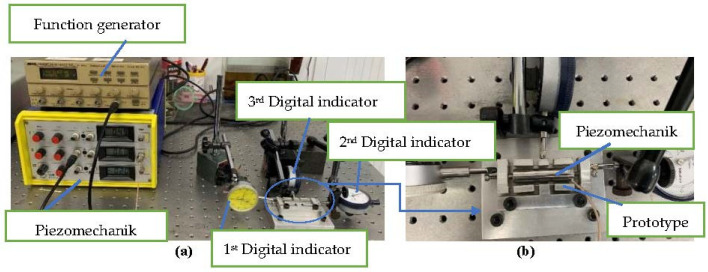
(**a**) The experimental set-up. (**b**) Larger view of the bridge-type compliant mechanism.

The optimal parameters *x*1*y*1*z*1*t*1*w*1 were utilized to manufacture a prototype bridge-type compliant mechanism. The mechanism was fabricated by the use of an electrical wire machine. The experiment was set up as shown in Figure 10a, and a larger view of the mechanism is shown in Figure 10b. The displacement of 0.01 mm was input by piezomechanik, and was measured by the first and then the second digital indicator. The function generator created a frequency function which was transmitted to the piezomechanik GmbH, and then the piezomechanik. The output displacement was measured using the third digital indicator.

The experimental results measured an output displacement of 0.6536 mm, and a displacement amplification ratio of 65.36. The optimal result and the experiment result were in good agreement, with a deviation of 5.97%, as depicted in Table 10. These values are better than those obtained in previous studies [3,7,8,9,10].

## 5. Conclusions

In this study, we have analyzed the results of design dimensions of the flexure joint and linked size on the displacement and stress of the bridge-type compliant mechanism, primarily based on the FEA in ANSYS. The FEA effects have proven that the design variables strongly impacted displacement and pressure. The design problems changed with the aid of using S/N evaluation, ANOVA, RE, and surface plot. All the results seem to be in great agreement. The expected values of GRG of the ANN approach have additionally confirmed the problem. The evidence has shown that the errors of the expected values of GRG with the ANN approach compared with those of the GRA approach were much less than 4%. It was found that the optimum values for displacement and pressure were 0.6951 mm and 78.834 MPa, respectively. The optimal values after investigated in this study were additionally confirmed by the experiments. All the effects were in good agreement, with an error of much less than 6%. The optimal magnification ratio was acquired as 65.36 times with the design variables (*x* = 5 mm, *y* = 0.4 mm, *z* = 0.7 degrees, *t* = 0 mm, and *w* = 4 mm, respectively).

## Figures and Tables

**Table 1:** Selected factors with their levels.

Parameters		Unit	Their Levels		
			First	Second	Third
Inlet frame length	*x*	mm	5	10	15
Flexure hinge thickness	*y*	mm	0.4	0.6	0.8
Two-flexure hinge distance	*z*	degrees	0.7	1.0	1.3
Fillet radius	*t*	mm	0.0	0.2	0.4
Flexure hinge width	*w*	mm	4	6	8

**Table 2:** Output response (including orthogonal arrays and numerical results with S/N).

Test No. (Trial)	*x*	*y*	*z*	*t*	*w*	Deformation (mm)	Stress (MPa)
1	5	0.4	0.7	0	4	0.6951	78.834
2	5	0.4	1	0.2	6	0.6378	92.646
3	5	0.4	1.3	0.4	8	0.5693	105.044
4	5	0.6	0.7	0.2	8	0.5384	92.330
5	5	0.6	1	0.4	4	0.5546	86.350
6	5	0.6	1.3	0	6	0.4725	90.349
7	5	0.8	0.7	0.4	6	0.3875	69.767
8	5	0.8	1	0	8	0.3541	64.184
9	5	0.8	1.3	0.2	4	0.3668	65.720
10	10	0.4	0.7	0	4	0.7153	116.280
11	10	0.4	1	0.2	6	0.6436	99.901
12	10	0.4	1.3	0.4	8	0.5855	96.782
13	10	0.6	0.7	0.2	8	0.4823	96.080
14	10	0.6	1	0.4	4	0.4977	91.180
15	10	0.6	1.3	0	6	0.4702	84.826
16	10	0.8	0.7	0.4	6	0.3935	82.673
17	10	0.8	1	0	8	0.3654	75.989
18	10	0.8	1.3	0.2	4	0.3755	76.678
19	15	0.4	0.7	0	4	0.7275	80.981
20	15	0.4	1	0.2	6	0.6502	84.490
21	15	0.4	1.3	0.4	8	0.6028	78.877
22	15	0.6	0.7	0.2	8	0.4923	92.956
23	15	0.6	1	0.4	4	0.5112	94.270
24	15	0.6	1.3	0	6	0.4676	86.395
25	15	0.8	0.7	0.4	6	0.4018	97.780
26	15	0.8	1	0	8	0.3714	84.367
27	15	0.8	1.3	0.2	4	0.3811	99.880

**Table 3:** For displacement and stress, the larger displacement is better and the smaller stress is better, and *Δ_oi_*(1) and *Δ_oi_*(2) show value deviation.

Trial Test No.	$D_{i}^{*}$ (1)	$D_{i}^{*}$ (2)	*Δ_oi_*(1)	*Δ_oi_*(2)
1	0.9055	0.1422	0.0945	0.8578
2	0.7598	0.4537	0.2402	0.5463
3	0.5763	0.2157	0.4237	0.7843
4	0.4936	0.4597	0.5064	0.5403
5	0.5370	0.5745	0.463	0.4255
6	0.3171	0.4978	0.6829	0.5022
7	0.0894	0.8928	0.9106	0.1072
8	0.0000	1.0000	1.0000	0.0000
9	0.0340	0.9705	0.966	0.0295
10	0.9673	0.0000	0.0327	1.0000
11	0.7753	0.3144	0.2247	0.6856
12	0.6197	0.3743	0.3803	0.6257
13	0.3433	0.3877	0.6567	0.6123
14	0.3846	0.4818	0.6154	0.5182
15	0.3109	0.6038	0.6891	0.3962
16	0.1055	0.6451	0.8945	0.3549
17	0.0303	0.7734	0.9697	0.2266
18	0.0573	0.7602	0.9427	0.2398
19	1.0000	0.6776	0.0000	0.3224
20	0.7930	0.6102	0.2070	0.3898
21	0.6660	0.7180	0.3340	0.2820
22	0.3701	0.4477	0.6299	0.5523
23	0.4207	0.4225	0.5793	0.5775
24	0.3040	0.5737	0.6960	0.4263
25	0.1277	0.3551	0.8723	0.6449
26	0.0463	0.6126	0.9537	0.3874
27	0.0723	0.3148	0.9277	0.6852

**Table 4:** The rank of GRG and the predicted GRG of ANN.

Trial No.	GRC (*γ_i_*(1))	GRC (*γ_i_*(2))	GRG (*ψ_i_*)	Rank	S/N of GRG	Predicted GRG of ANN	Error
1	0.841	0.3682	0.7406	2	−2.73712	0.729699	0.010901463
2	0.6755	0.4779	0.5770	9	−4.6673	0.584299	−0.007298969
3	0.5413	0.3893	0.4655	20	−6.79307	0.457453	0.008046562
4	0.4968	0.4806	0.4887	16	−6.20221	0.489654	−0.000953627
5	0.5192	0.5402	0.5297	11	−5.44135	0.534481	−0.004781168
6	0.4227	0.4989	0.4607	22	−6.71628	0.461515	−0.000814538
7	0.3545	0.8235	0.5883	8	−4.6074	0.588342	−4.24 × 10^−5^
8	0.3333	1.0000	0.6656	3	−3.54029	0.665251	0.000448659
9	0.3411	0.9443	0.6418	4	−4.11459	0.622688	0.019111683
10	0.9386	0.3333	0.6369	5	−3.23449	0.689089	−0.052289264
11	0.6899	0.4217	0.5562	10	−5.46608	0.532962	0.023237886
12	0.568	0.4442	0.5063	14	−5.85577	0.509579	−0.003279069
13	0.4323	0.4495	0.4409	25	−7.19254	0.436891	0.004008545
14	0.4483	0.4911	0.4696	19	−6.97332	0.448058	0.021542182
15	0.4205	0.5579	0.4890	15	−6.62479	0.466402	0.022597861
16	0.3586	0.5849	0.4714	18	−6.66318	0.464345	0.007055262
17	0.3402	0.6881	0.5136	12	−5.83493	0.510803	0.002797351
18	0.3466	0.6759	0.5107	13	−5.91761	0.505964	0.004836211
19	1.0000	0.6080	0.8046	1	−2.1881	0.777311	0.027288788
20	0.7072	0.5619	0.6348	6	−3.79827	0.645783	−0.010982537
21	0.5995	0.6394	0.6194	7	−4.15325	0.619923	−0.000523041
22	0.4425	0.4751	0.4587	23	−6.66189	0.464414	−0.005613602
23	0.4633	0.464	0.4636	21	−6.60591	0.467417	−0.003816678
24	0.4181	0.5398	0.4788	17	−6.34858	0.481472	−0.002671981
25	0.3644	0.4367	0.4004	26	−7.90211	0.402619	−0.002219156
26	0.3439	0.5634	0.4533	24	−6.68759	0.463042	−0.009741989
27	0.3502	0.4219	0.3859	27	−7.32292	0.430382	−0.04448199

**Table 5:** Responses for S/N ratio (GRG).

Levels	*x*	*y*	*z*	*t*	*w*
First	−4.944	−4.328	−5.290	−4.884	−5.015
Second	−5.886	−6.467	−5.420	−5.765	−5.806
Third	−5.881	−5.916	−6.002	−6.063	−5.890
Delta	0.942	2.140	0.712	1.179	0.875
Rank	3	1	5	2	4

**Table 6:** Outcomes of analyzing mean values.

Levels	*x*	*y*	*z*	*t*	*w*
First	0.5759	0.6157	0.5731	0.5826	0.5589
Second	0.5174	0.4755	0.5105	0.5216	0.5404
Third	0.5124	0.5145	0.5222	0.5016	0.5064
Delta	0.0635	0.1402	0.0509	0.0810	0.0525
Rank	3	1	5	2	4

**Table 7:** Details of ANOVA results (GRG).

Sources	DF	Seq + SS	Contributions	AdjSS	SeqMS	F-Values	*p*-Values
*Reg*	9	0.098263	96.61%	0.098263	0.010918	33.16	0.000
*x*	1	0.003510	4.38%	0.002514	0.003510	10.66	0.005
*y*	1	0.015414	14.84%	0.006638	0.015414	46.82	0.000
*z*	1	0.002495	2.40%	0.004054	0.002495	7.58	0.014
*t*	1	0.008349	8.04%	0.000824	0.008349	25.36	0.000
*w*	1	0.004259	4.10%	0.005381	0.004259	12.94	0.002
*x* × *x*	1	0.001945	1.87%	0.001945	0.001945	5.91	0.026
*y* × *y*	1	0.017658	17.00%	0.017658	0.017658	53.64	0.000
*x* × *y*	1	0.042918	41.32%	0.042918	0.042918	130.37	0.000
*y* × *t*	1	0.001715	1.65%	0.001715	0.001715	5.21	0.036
Error	17	0.005597	3.39%	0.005597	0.000329		
Total	26	0.103860	100.00%				

*R*-*sq* = 96.61%, *R*-*sq* (adj) = 94.76%, *R*-*sq* (pred) = 92.92%.

**Table 8:** The results of the statistical analysis of GRG.

RMSE	MSE	MAPE	*R*-Square
0.017181371	0.000295199	2.109164529	0.998937275

**Table 9:** Comparison of the predicted and optimal values between methods.

Method	TM	ANN	RE
Predicted value of GRG	0.7650	0.7671	0.768
Optimal value of GRG	0.7406	0.7406	0.7406
% Error	3.2	3.46	3.57

**Table 10:** Comparison of the optimal value with the experimental results.

Output	Combination Parameters	Di (mm)	Magnification Ratio	Stress (MPa)
Optimal value	*x*1*y*1*z*1*t*1*w*1	0.6951	69.22	78.834
Experiment value		0.6536	65.36	-
% Error		5.97	5.97	
